# A222 PATTERNS OF SPECIALIST HEALTHCARE DELIVERY AMONG INFLAMMATORY BOWEL DISEASE PATIENTS IN RESPONSE TO THE COVID-19 PANDEMIC IN ONTARIO

**DOI:** 10.1093/jcag/gwae059.222

**Published:** 2025-02-10

**Authors:** L J Nguyen, V Huang, P Habashi, P Tandon

**Affiliations:** Lunenfeld-Tanenbaum Research Institute, Toronto, ON, Canada; Sinai Health, Toronto, ON, Canada; Lunenfeld-Tanenbaum Research Institute, Toronto, ON, Canada; University Health Network, Toronto, ON, Canada

## Abstract

**Background:**

The inflammatory bowel diseases (IBD), which comprise Crohn’s disease (CD) and ulcerative colitis (UC) are chronic conditions that can lead to significant disease complications. Access to specialist care has been shown to reduce the risk of surgery. However, due to restrictions during the COVID-19 pandemic, healthcare providers had to quickly pivot to virtual healthcare delivery.

**Aims:**

Our aims were to characterize patterns of virtual care by specialists during the pandemic and whether these patterns differed between regions with high versus low access to gastroenterologists.

**Methods:**

We used administrative databases at ICES, Ontario to identify the study cohort. All individuals aged 18 years or older who had an IBD diagnosis at any point between April 1, 2016 and March 31, 2021 were identified in the Ontario Crohn’s and Colitis Cohort and linked to the Ontario Health Insurance Plan (OHIP) and the ICES Physician’s Database (IPDB) to ascertain specialists visits for IBD. Tariff codes were used to categorize each outpatient IBD specialist visit as in-person or virtual (by phone or video). We calculated the rate of IBD specialist visits per 100 IBD capita for each quarter and stratified these by geographic regions that had low versus high access to gastroenterologists.

**Results:**

There were 95,879 adult individuals living with IBD in Ontario at the beginning of the study. Figure 1 shows the quarterly rates of in-person and virtual IBD specialist visits four years prior to the COVID-19 pandemic and one year after its start during the first quarter of 2020. Prior to the pandemic IBD specialist visits were almost all in-person and there was a gap in rates between regions with low and high access to gastroenterologists. There was also a slight downward trend in rates of IBD specialist visits in all regions in the few years leading up to the pandemic. During the first quarter of 2020, there was an abrupt transition where the rates of in person IBD specialist visits plummeted, as rates of virtual IBD specialist visits rapidly ascended approaching rates of in-person IBD specialists visits pre-pandemic. The total rate of in-person and virtual IBD specialist visits increased during the pandemic compared to pre-pandemic rates.

**Conclusions:**

Although the COVID-19 pandemic posed barriers in accessing in person healthcare, the rapid adoption of virtual care helped to compensate for this limitation. Virtual care was effectively implemented in both regions with low and high specialist access, likely facilitated by inclusion of services over the phone. The advent of virtual care increased the rate of total IBD specialist services in all regions during the pandemic. The sustainability of virtual care remains to be seen after the pandemic and after reduction of payment for phone services.

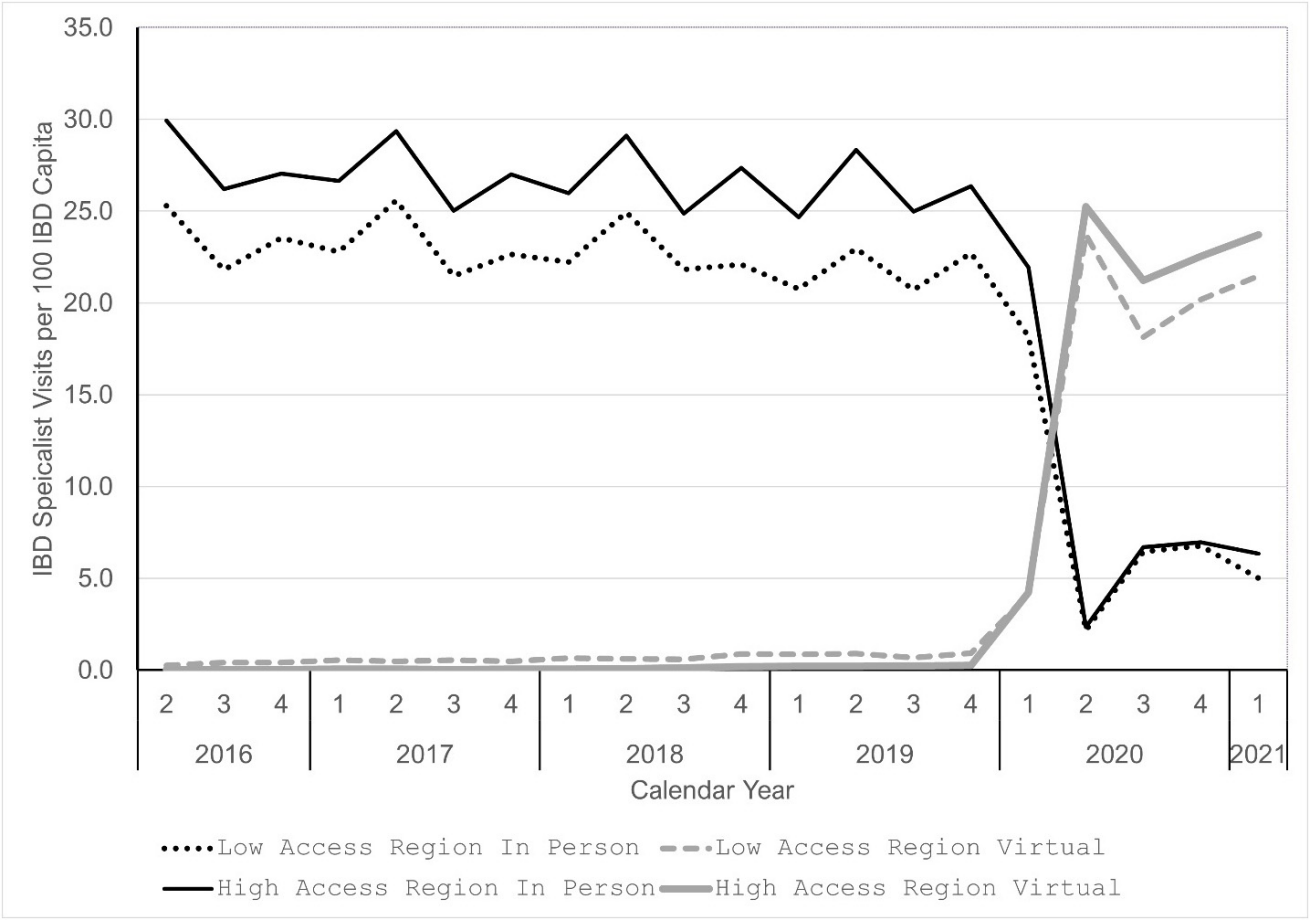

Figure 1. Rates of IBD specialist visits per 100 IBD capita prior to and during the COVID-19 pandemic stratified by type of visit and by geographic region

**Funding Agencies:**

CCC

